# Nonsteroidal Anti-Inflammatory Drug Injections versus Steroid Injections in the Management of Upper and Lower Extremity Orthopedic Conditions: A Systematic Review with Meta-Analysis

**DOI:** 10.3390/jcm13041132

**Published:** 2024-02-17

**Authors:** Hye Chang Rhim, Joseph Ruiz, Atta Taseh, Wilma Afunugo, Zack Crockett, Jason Schon, Xiaoyu Pan, Jaehyung Shin, Sean Schowalter, Ki-Mo Jang, David M Robinson

**Affiliations:** 1 Department of Physical Medicine and Rehabilitation, Harvard Medical School/Spaulding Rehabilitation Hospital, Boston, MA 02115, USA; hrhim@mgh.harvard.edu (H.C.R.); jruiz8@mgb.org (J.R.); wafunugo@mgb.org (W.A.); zcrockett@mgb.org (Z.C.); jschon@mgb.org (J.S.); drobinson22@mgb.org (D.M.R.); 2 Foot & Ankle Research and Innovation Lab (FARIL), Department of Orthopaedic Surgery, Harvard Medical School, Massachusetts General Hospital, Boston, MA 02115, USA; ataseh@mgh.harvard.edu (A.T.); jshin366@gatech.edu (J.S.); 3 Department of Epidemiology, Harvard T.H. Chan School of Public Health, Boston, MA 02115, USA; xiaoyu_pan@hsph.harvard.edu; 4 Department of Nutrition, Harvard T.H. Chan School of Public Health, Boston, MA 02115, USA; 5 Department of Sports Medicine, Mount Sinai School of Medicine, New York, NY 10029, USA; 6 Department of Orthopaedic Surgery, Anam Hospital, Korea University College of Medicine, Seoul 02841, Republic of Korea

**Keywords:** ketorolac, triamcinolone, methylprednisolone, shoulder impingement syndrome, knee osteoarthritis, hip osteoarthritis, trigger finger

## Abstract

Background: Although corticosteroid injections are an effective treatment for musculoskeletal pathologies, they may not be suitable for all patients. The purpose of this systematic review was to compare clinical outcomes between patients who received NSAID and corticosteroid injections for various orthopedic conditions. Methods: Medline, Embase, Web of Science, and Cochrane Central Register of Controlled Trials were searched, and meta-analyses were performed using a random-effects model for outcomes presented in three or more studies. Other studies were qualitatively analyzed. Results: A total of 28 articles with 2113 patients were included. A meta-analysis of five studies in patients with shoulder impingement syndrome demonstrated that there was no significant difference in the pain visual analogue scale (VAS) between subacromial NSAID injections and corticosteroid injections at 1 month [weighted mean difference (WMD) −0.244; 95% CI, −1.232 to 0.745; I^2^, 94.5%]. For patients with knee osteoarthritis, a meta-analysis of three studies demonstrated that there was no significant difference between intraarticular NSAID injections and corticosteroid injections in pain VAS at 1 month (WMD 0.754; 95% CI, −0.413 to 1.921; I^2^, 90.2%) and 3 months (WMD−0.089; 95% CI, −0.345 to 0.166; I^2^, 0%). A review of the studies assessing pain outcomes for hip osteoarthritis, adhesive capsulitis, and plantar fasciitis showed no significant differences between the NSAID and corticosteroid groups. Conclusion: NSAID injections may be safe and effective alternatives to steroid injections, especially in shoulder impingement syndrome and knee osteoarthritis.

## 1. Introduction

Musculoskeletal disorders represent a significant challenge in the domains of sports medicine and orthopedics, affecting a broad range of patients. The management of these conditions requires a multifaceted approach that seeks to alleviate pain, restore functionality, and improve the overall quality of life. Among the plethora of treatment modalities, corticosteroid injections have long been a mainstay in the therapeutic arsenal [1].

Steroid injections are employed across a wide array of orthopedic conditions such as osteoarthritis [2], shoulder impingement [3], adhesive capsulitis [4], greater trochanteric pain syndrome [5], and trigger finger [6], demonstrating pain relief and functional improvement. However, the administration of steroids is not without its drawbacks. Local and systemic adverse events have been associated with their use, including reduced bone mineral density [7], tendon rupture or atrophy [8,9], osteonecrosis [10,11,12], and hyperglycemia [13]. These concerns are particularly significant in the context of frequent and prolonged use, necessitating a careful balancing act between the potential benefits and inherent risks.

Oral nonsteroidal anti-inflammatory drugs (NSAIDs) also represent another prevalent treatment option, and some studies have demonstrating similar efficacy between oral NSAIDs and steroid injections [14,15]. However, the use of oral NSAIDs has also been linked to serious side effects, such as gastrointestinal (GI) bleeding, cardiovascular complications, and renal impairment, which may limit their applicability for some patients [16,17]. This has spurred interest in exploring NSAID injections as an alternative therapeutic option, which may minimize systemic side effects [18] while providing significant pain relief and functional improvement.

Consequently, several comparative studies between NSAID injections and steroid injections have emerged over the past two decades. However, the findings have been mixed, and the question remains whether NSAID injections can serve as viable alternatives, particularly when steroid injections are contraindicated in selected patients. Up to date, one systematic review compared intraarticular NSAID and corticosteroid injections for musculoskeletal pain but only included four studies [19], and another systematic review focused on shoulder impingement syndrome [20]. There has not been a review that has comprehensively overviewed comparisons between NSAID injections and steroid injections in diverse musculoskeletal conditions. Therefore, the present systematic review aimed to synthesize the available literature that compared NSAID injections with steroid injections in various upper and lower extremity conditions to guide clinicians in evidence-based decision-making and to propose future research directions.

## 2. Materials and Methods

### 2.1. Systematic Review Registration

The protocol for this systematic review was registered at the International Platform of Registered Systematic Review and Meta-Analysis Protocols (INPLASY202380003).

### 2.2. Search Strategy and Selection Criteria

We searched Medline, Embase, Web of Science, the Cochrane Center Register of Controlled Trials for randomized clinical trials (RCTs) and comparative studies that compared NSAID injections with steroid injections in upper and lower extremity orthopedic conditions. The literature search was conducted per the Preferred Reporting Items for Systematic Reviews and Meta-Analyses (PRISMA) guidelines and included all studies published from the database inception through August 2023 [21]. A librarian affiliated with the authors’ institution was consulted to build search terms (Supplemental Table S1). A manual search was also conducted using reference lists of relevant articles. RCTs or comparative studies that compared outcomes between NSAID injections and steroid injections in upper and lower extremity conditions were eligible for inclusion. Studies that involved intramuscular or intravenous injections, investigated postoperative pain control or spine conditions, and included rheumatologic disorders were excluded.

### 2.3. Data Extraction and Quality Assessment

Two authors independently assessed each study from the initial search and conducted data extraction. Variables extracted from each article included the country where the study was conducted, study design, pathology, population, types and dosages of NSAIDs and steroids, injection method, post-injection protocol, rehabilitation, outcome measures, follow-up durations, main findings, and adverse events. Two authors evaluated the risk of bias with the revised Cochrane risk-of-bias tool for randomized trials for RCTs [22] and the Newcastle–Ottawa Scale (NOS) for non-RCTs [23]. Discrepancies between the two authors were resolved by discussion with a third author.

### 2.4. Statistical Analysis

We planned to perform a meta-analysis for outcomes that were reported in 3 or more studies (RCTs), and as a result, meta-analyses were performed for shoulder impingement syndrome and knee osteoarthritis. The mean and standard deviation (SD) of outcome measures from these conditions after NSAID and steroid injections were extracted for statistical analysis. Heterogeneity was assessed with the Q and I^2^ statistics [24]. We conducted random-effects pairwise meta-analyses to account for clinical heterogeneity across the studies including patient and injection characteristics as random-effects methods allow the modelling of differences among studies and tend to offer more conservative results [25]. Therefore, a fixed-effect model was not used even when I^2^ < 50%. The weighted mean difference (WMD) between the NSAID injection and steroid injection was used as a measure of the effect size for the pain visual analogue scale (VAS) for shoulder impingement syndrome and for knee osteoarthritis. Outcomes that were collected at 3weeks were merged with the 1-month outcome for the meta-analysis. For the study that compared ketorolac with triamcinolone and betamethasone, outcomes with triamcinolone were used for meta-analysis [26]. Publication bias was not assessed because the included number of studies was less than ten. STATA Version 16 (StataCorp, LLC, College Station, TX, USA) was used for all analyses.

## 3. Results

### 3.1. Eligible Studies and Characteristics of Included Studies

**Figure 1:**
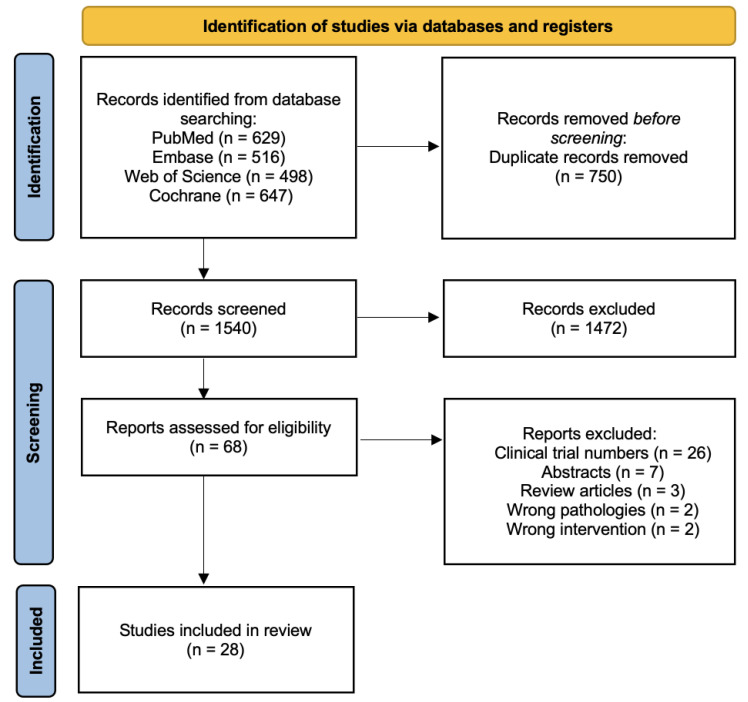
PRISMA (Preferred Reporting Items for Systematic Meta-Analyses) diagram showing selection of articles for this meta-analysis.

The initial search of published articles yielded 2290 studies. Furthermore, 750 duplicate articles were removed. Of the 1540 articles reviewed, 1472 articles met the exclusion criteria based on a review of titles and abstracts. Sixty-eight records were reviewed in full, and 40 studies were excluded because they were clinical trial numbers without published studies (*n* = 26), abstracts (*n* = 7), review articles (*n* = 3), studies including pathologies that did not meet our inclusion criteria (*n* = 2), and studies including oral NSAIDs (*n* = 2). The resulting 28 articles with a total number of 2113 patients identified in the search were included in this review. The review process is outlined in the PRIMSA flow diagram (Figure 1).

Among the studies that investigated a single pathology, shoulder impingement syndrome was most widely studied (*n* = 11), followed by knee osteoarthritis (*n* = 7), trigger finger (*n* = 3), hip osteoarthritis (*n* = 2), adhesive capsulitis (*n* = 1), plantar fasciitis (*n* = 1), carpal tunnel syndrome (*n* = 1), and de Quervain’s tenosynovitis (*n* = 1). Two studies investigated multiple pathologies. One of these studies [27] focused on shoulder pathologies including adhesive capsulitis (50%), rotator cuff syndrome (40%), and shoulder impingement syndrome (10%), and the other study [28] focused on soft tissue injuries including bicipital tendinitis (21.7%), de Quervain’s tenosynovitis (31.7%), knee bursitis (4.2%), lateral epicondylitis (15%), myofascial pain syndrome (2.5%), patellar tendinitis (2.5%), and plantar fasciitis (22.5%).

For NSAID injections, ketorolac was used in 19 studies, tenoxicam in 4 studies, lornoxicam in 2 studies, and diclofenac in 2 studies. For steroid injections, triamcinolone was used in 19 studies, methylprednisolone in 9 studies, and betamethasone in 2 studies (1 study used both triamcinolone and betamethasone as a comparator [26]). Other characteristics of the included studies are summarized in Table 1, and the main findings from each study are summarized in Table 2.

Among 22 RCTs, 16 trials reported whether they received funding or not [26,28,29,30,31,32,33,34,35,36,37,38,39,40,41,42], and none of them were industry-sponsored studies. 

**Table 1:** Characteristics of included studies comparing nonsteroidal anti-inflammatory drug injections with steroid injections in upper and lower extremity orthopaedic conditions.

Author	Country	Study Design	Pathology	Population	NSAID	Steroid	Injection Method	Post-injection Protocol	Rehabilitation	Outcome Measures	Follow-up Duration	Adverse Events
Abolhasani 2019 [26]	Iran	RCT	Shoulder impingement syndrome	Ketorolac: 35 patients; 16 M, 19 F Triamcinolone: 35 patients; 17 M, 18 F Betamethasone: 35 patients; 15 M, 20 F Total mean age 37.38 ± 9.45 yrs	Ketorolac: 2 × 1 mL ampoule + 2% lidocaine 4 mL + normal saline 4 mL, total 10 mL	Triamcinolone: 2 × 1 mL ampoule + 2% lidocaine 4 mL + normal saline 4 mL, total 10 mL Betamethasone LA: 2 × 1 mL ampoule + 2% lidocaine 4 mL + normal saline 4 mL, total 10 mL	Posterior approach	NA	NA	VAS; OSS	2, 4, and 6 wks	NA
Ahn 2015 [43]	South Korea	Retrospective comparative study	Adhesive capsulitis	Ketorolac + capsular distension: 64 patients; 11 M, 53 F; mean age 54.63 ± 5.62 yrs; mean duration 7.43 ± 2.32 mo Triamcinolone: 57 patients; 8 M, 49 F; 55.23 ± 4.69 yrs; 7.23 ± 1.94 mo	Ketorolac + capsular distension: 0.5% lidocaine 19 mL + ketorolac 30 mg (1 mL) + capsular distension.	Triamcinolone: 0.5% lidocaine 4 mL + triamcinolone 40 mg (1 mL)	Posterior approach (ultrasound-guided)	Limit shoulder motion for five to ten minutes after injection; second injection offered if less than 50% VAS improvement in two weeks.	Home exercise program	SPADI; VAS; passive ROM	1, 3, and 6 mo	NSAID: two cases of dizziness and transient muscle weakness Steroid: two cases of steroid-induced synovitis Both groups: no other severe complications
Aksakal 2017 [44]	Turkey	RCT	Shoulder impingement syndrome	Lornoxicam: 35 patients; 14 M, 21 F; mean age 53.0 ± 5.5 yrs; mean duration 2–7 wks Betamethasone: 35 patients; 12 M, 23 F; mean age 53.0 ± 5.3 yrs; mean duration 2–8 wks	Lornoxicam: 2 mL (8 mg)	Betamethasone: 1 mL (6.43 mg betamethasone dipropionate, 2.63 mg betamethasone sodium phosphate	Posterior approach (blind)	NA	NA	CMS; UCLA score	2, 4, and 6 wks	NA
Bayat 2018 [45]	Iran	RCT	Knee osteoarthritis (KL grade 2 or 3)	Ketorolac: 19 patients; 1 M, 18 F; mean age 59.9 ± 8.6 yrs; mean duration 3.2 ± 2.6 yrs Triamcinolone: 19 patients; 3 M, 16 F; mean age 61.1 ± 5 yrs; mean duration 4.3 ± 1.9 yrs	Ketorolac: ketorolac 30 mg+ 2% lidocaine 1 mL at baseline	Triamcinolone: triamcinolone 40 mg + 2% lidocaine 2% at baseline	Lateral midpatellar approach in an extended knee using a 22G needle	NA	Exercise therapy—multi-angle isometric exercises along with hamstring stretching three times per day; closed-chain isotonic exercises was started after the first month	WOMAC; Lequesne scale; VAS	4, and 12 wks	NA
Bellamy 2016 [46]	USA	RCT	Knee osteoarthritis (mean KL grade 3)	Ketorolac: 16 patients; 7 M, 9 F; mean age 53 yrs Triamcinolone: 20 patients; 9 M, 11 F; mean age 65 yrs	Ketorolac: 2 mL (toradol 30 mg) in 8 mL of bupivacaine hydrochloride (0.5%) without epinephrine	Triamcinolone acetonide: 2 mL of kenalog 40 mg/mL in 8 mL of bupivacaine hydrochloride (0.5%) without epinephrine	Superolateral approach (ultrasound-guided)	NA	NA	VAS; WOMAC; KS score; Tegner/Lysholm Knee Scoring Scale; Short Form 36; UCLA activity score	2 wks, 6 wks, 3 mo, and 6 mo	NA
Cift 2015 [29]	Turkey	RCT	Shoulder impingement syndrome	Tenoxicam: 20 patients; 10 M, 10 F; mean age 45.3 ± 8.8 yrs Methylprednisolone: 20 patients; 8 M, 12 F; mean age 46.5 ± 11 yrs	Tenoxicam: 20 mg three times by weekly intervals	Methylprednisolone: 40 mg once	Posterior arthroscopy portal (used for the needle entry point; sitting position)	NA	All patients underwent home exercise program including gravity-assisted distraction oscillatory pendulum exercise	VAS; active ROM; DASH	6 wks and 1 yr	There were no major complications, but patients in the tenoxicam group complained of a moderate burning-like sensation during injection, and two patients had temporary hypotension.
Gondal 2021 [30]	Pakistan	RCT	Knee osteoarthritis (KL grade 2 or 3)	Ketorolac: 40 patients; 14 M, 26 F; mean age 55.6 ± 9.2 yrs Triamcinolone: 40 patients; 16 M, 24 F; mean age 57.2 ± 8.30 yrs	Ketorolac: 30 mg + 5 mL lidocaine and 2.5 mL (25 mg) sodium hyaluronate	Triamcinolone: 80 mg + 5 mL lidocaine and 2.5 mL (25 mg) sodium hyaluronate	Ultrasound-guided	NA	NA	VAS; WOMAC	1 wk, 1 mo, and 3 mo	NA
Goyal 2022 [31]	India	RCT	Shoulder impingement syndrome	Ketorolac: 34 patients; 14 M, 20 F; mean age 51.57 ± 13.22 yrs; mean duration 4.86 ± 1.3 mo Methylprednisolone: 33 patients; 10 M, 23 F; mean age 52.7 ± 11.81 yrs; mean duration 5.28 ± 1.1 mo	Ketorolac: 60 mg + 2% lignocaine 5 mL	Methylprednisolone: 40 mg + 2% lignocaine 5 mL	Posterior – lateral approach (palpation-guided)	Injections repeated at four weeks if symptoms persisted.	Rotator cuff strengthening exercises; capsular stretching exercises; shoulder ROM exercises	VAS; SPADI; ROM (flexion, abduction, ER, IR)	1 mo and 3 mo	NA
Guner 2013 [32]	Turkey	RCT	Plantar fasciitis	Total: 61 patients (tenoxicam 31, methylprednisolone 30); 14 M, 47 F; mean age 41.4 ± 12.23 yrs	Tenoxicam: 1 mL of tenoxicam (20 mg/2 mL) + 2% lidocaine 1 mL	Methylprednisolone: 1 mL injection of 40 mg of methylprednisolone acetate + 2% lidocaine 1 mL	Medial approach (palpation-guided)	Not allowed to move ten minutes after injection; activity restriction for 4 weeks	Stretching; strengthening exercises	VAS; RM	6 and 12 mo	NA
Jami 2020 [33]	Iran	RCT	Trigger finger	Diclofenac: 42 patients; 10 M, 32 F; mean age 52 ± 9 yrs Methylprednisolone: 42 patients; 11 M, 31 F; mean age 53 ± 7 yrs	Diclofenac: 12.5 mg diclofenac sodium injection	Methylprednisolone: 20 mg methylprednisolone acetate injection	Tendon sheath and 8 mm above the MP	NA	NA	Quinnell grading	1, 3, and 6 wks; 3, 6, and 12 mo	NA
Jurgensmeier 2021 [34]	USA	RCT	Hip and knee osteoarthritis (KL grade 2 or higher)	Total: 120 patients (ketorolac 59, triamcinolone 61); 77 F, 43 M; mean age 65.28 ± 12.6 yrs	Ketorolac: 30 mg + 1% lidocaine 5 mL + 0.5% ropivacaine 5 mL	Triamcinolone: 80 mg + 1% lidocaine 5 mL + 0.5% ropivacaine 5 mL	Ultrasound-guided	NA	NA	HOOS; KOOS; VAS; PROMIS global health score	1 wk, 1 mo, and 3 mo	Ketorolac: one patient with headache and nausea, which resolved the next day; GI bleeding in a patient who had increased warfarin dose approximately two months after ketorolac injection Triamcinolone: hyperglycemia in two diabetic patients during the first week of post-injection; nausea, hypertension, and flare of temporal arteritis were each recorded once
Karimzadeh 2023 [35]	Iran	RCT	Carpal tunnel syndrome (mild to moderate)	Ketorolac: 21 patients; 4 M, 17 F; mean age 50.71 ± 9.92 yrs; mean duration 16.62 ± 0.43 mo Triamcinolone: 22 patients; 6 M, 16 F; mean age 53.05 ± 6.80 yrs; mean duration 16.23 ± 0.57 mo	Ketorolac: 1 mL of 30 mg/mL + 2% lidocaine 0.5 mL	Triamcinolone: 1 mL of 40 mg/mL + 2% lidocaine 0.5 mL	Ultrasound-guided	Relative rest for 24 h; recommended to apply cold compress for ten minutes three times daily; allowed to take acetaminophen 500 mg (without codeine) every 4 to 8 h; not allowed to take other pain-relieving medications such as NSAIDs, dietary supplements, or vitamins for 1 week after the injection.	Allowed to do light to moderate physical activity gradually increase it at their own pace	VAS; BCTQ; electrodiagnostic findings; patient satisfaction; complication	12 wks	Seven patients from both groups experienced adverse events such as warm sensation, stiffness, and heaviness at the injection site without significant group difference
Karthikeyan 2010 [47]	England	RCT	Shoulder impingement syndrome	Tenoxicam: 31 patients; 16 M, 15 F; mean age 58.0 ± 9.8 yrs; mean duration 8 (range 2–12) mo Methylprednisolone: 27 patients; 16 M, 11 F; mean age 60.0 ± 13.0 yrs; mean duration 10 (range 2–12) mo	Tenoxicam: 20 mg + 1% lignocaine 5 mL	Methylprednisolone: 40 mg + 1% lignocaine 5 mL	Anterolateral approach (reduction in pain of at least 50% with Neer’s test ten minutes after injection to confirm accurate placement)	Advised to take simple analgesia if needed but to avoid any preparation containing NSAIDs	Standardized outpatient physiotherapy for all patients	CMS; DASH; OSS	2, 4, and 6 wks	NA
Kim 2021 [36]	South Korea	RCT	Shoulder impingement syndrome	Ketorolac: 30 patients; 19 M, 11 F; mean age 66 ± 6.0 yrs; mean duration 8.8 ± 7.2 mo Triamcinolone: 30 patients; 22 M, 8 F; mean age 68.8 ± 6.0 yrs; mean duration 7.4 ± 5.5 mo	Ketorolac: 1 mL of 30 mg/mL + 2% lidocaine 1 mL	Triamcinolone: 1 mL of 40 mg/mL + 2% lidocaine 1 mL	Palpation-guided	Prescribed with acetaminophen	Home exercise program	VAS; ASES; UCLA score; patient satisfaction	3, 6, 12 wks	Triamcinolone: one case of uncontrolled diabetes and two cases of facial flushing
Kwon 2017 [48]	South Korea	Retrospective comparative study	Shoulder impingement syndrome	Ketorolac: 20 patients; 18 M, 2 F; median age 54.45 (42–68); mean duration 12.20 ± 25.66 wks Triamcinolone: 20 patients; 16 M, 4 F; median age 56.25 (42–69); mean duration 7.25 ± 12.72 wks	Ketorolac: 60 mg + 1% lidocaine 5 mL	Triamcinolone: 40 mg + 1% lidocaine 5 mL	Ultrasound-guided	Advised not to take additional NSAID	Home exercise program	VAS; CMS; ROM (forward flexion, IR, ER)	1 mo and 3 mo	NA
Leow 2018 [37]	Singapore	RCT	Trigger finger	Ketorolac: 59 patients; 23 M, 36 F Triamcinolone: 62 patients; 22 M, 40 F Total mean age of 60 yrs (range 44–87)	Ketorolac: 0.5 mL of 30 mg/mL + 1% lidocaine 1% 0.5 mL (10 mg/mL)	Triamcinolone: 0.5 mL of 10 mg/mL + 1% lidocaine 0.5 mL (10 mg/mL)	Intrasynovial and extrasynovial injection at the level of the A1 pulley	NA	NA	Severity; pain; A1 pulley tenderness and swelling; total active motion and flexion deformity; patient subjective responses; conversion to surgery	3, 6, 12 and 24 wks	NA
Matee 2021 [27]	Pakistan	Quasi-experimental study	Adhesive capsulitis (50%); rotator cuff syndrome (40%); shoulder impingement syndrome (10%)	Ketorolac: 36 patients; 26 M, 10 F; mean age 45 ± 10 yrs Methylprednisolone: 24 patients; 14 M, 10 F; mean age 55 ± 9 yrs	Ketorolac: 1 mL (30 mg) + 2% lignocaine 2 mL	Methylprednisolone: 1 mL (40 mg) + 2% lignocaine 2 mL	Posterior approach glenohumeral injection (blind)	Paracetamol (650 mg) and orphenadrine citrate (50 mg) twice a day, and local application of piroxicam gel 0.5% four times a day	Therapeutic exercises particular to disease	ROM (flexion, extension, abduction, IR, ER)	4 wks	NA
Min 2013 [38]	USA	RCT	Shoulder impingement syndrome	Ketorolac: 17 patients; 13 M, 4 F; mean age 39.6 ± 9.4 yrs Triamcinolone: 15 patients; 12 M, 3 F; mean age 39.1 ± 10.5 yrs	Ketorolac: 60 mg + 1% lidocaine 6 mL with epinephrine	Triamcinolone: 40 mg + 1% lidocaine 6 mL with epinephrine	Posterolateral approach (palpation-guided)	NA	NA	VAS; UCLA score; ROM (abduction and forward flexion)	4 wks	Triamcinolone: one case of a fainting episode that resolved in five minutes without intervention
Park 2015 [49]	South Korea	Retrospective comparative study	Hip osteoarthritis (KL grade 2 or 3)	Ketorolac: 12 M, 36 F; mean age 59.29 ± 8.78 yrs; mean duration 7.10 ± 2.25 mo Triamcinolone: 12 M, 38 F; mean age 58.24 ± 8.52 yrs; mean duration 6.66 ± 2.45 mo	Ketorolac: 30 mg (1 mL) + 0.5% lidocaine 14 mL	Triamcinolone: 40 mg (1 mL) + 0.5% lidocaine 14 mL	Ultrasound-guided	Patients were changed to acetaminophen or tramadol-acetaminophen if they were taking oral NSAIDs	All patients were continued on previous physical therapy	HHS; VAS	1, 3, and 6 mo	Ketorolac: four local adverse events described as mild, transient sensations of pain and heaviness in the injected joint
Shakeel 2012 [39]	Malaysia	RCT	Trigger finger (at least grade 2 by Quinnell)	Diclofenac: 50 patients; 18 M, 32 F; mean age 58 yrs (range 40–75) Triamcinolone: 50 patients; 12 M, 38 F; mean age 57 yrs (range 40–71)	Diclofenac: 0.5 mL of 12.5 mg	Triamcinolone: 0.5 mL of 20 mg	Targeting above the tendon and in the tendon sheath under the A1 pulley (palpation-guided)	NA	NA	Quinnell grading	3 wks and 3 mo	Diclofenac: two patients (4%) with continuous pain at the injection site; three patients (6%) with nodular swelling at the injection site; two patients (4%) with stiffness of the injected finger; one patient (2%) with recurrence of triggering painTriamcinolone: one patient (2%) with continuous pain at the injection site; nine patients (18%) with recurrence of triggering pain
Siddique 2021 [50]	Pakistan	RCT	Shoulder impingement syndrome	Ketorolac: 109 patients; 54 M, 55 F; mean age 39.09 ± 9.9 yrs Methylprednisolone: 109 patients; 63 M, 46 F; mean age 38.08 ± 8.61 yrs	Ketorolac: 60 mg + 2% lidocaine 1 mL	Methylprednisolone: 40 mg mixed + 2% lidocaine 1 mL	NA	NA	NA	VAS; CMS	4 wks	NA
Sindhupakorn 2020 [28]	Thailand	RCT	Soft tissue injuries; bicipital tendinitis (21.7%); de Quervain’s tenosynovitis (31.7%); knee bursitis (4.2%); lateral epicondylitis (15%); myofascial pain syndrome (2.5%); patellar tendinitis (2.5%); plantar fasciitis (22.5%)	Normal saline: 20 patients; 5 M, 15 F; mean age 54.2 ± 7.52 yrsKetorolac 30 mg: 20 patients; 9 M, 11 F; mean age 49.85 ± 9.75 yrs Ketorolac 60 mg: 20 patients; 8 M, 12 F; mean age 50.95 ± 7.54 yrs Triamcinolone 10 mg: 20 patients; 8 M, 12 F; mean age 48.1 ± 8.71 yrs Triamcinolone 20 mg: 20 patients; 5 M, 15 F; mean age 49.8 ± 10.22 yrs Triamcinolone 40 mg: 20 patients; 5 M, 15 F; mean age 52.15 ± 8.65 yrs	Ketorolac 30 mg: 1 mL (30 mg) + 2% xylocaine 2 mL Ketorolac 60 mg: 2 mL (60 mg) + 2% xylocaine 2 mL	Normal saline 0.9%: 1 mL + 2% xylocaine 2 mL Triamcinolone: 1 mL (10 mg) + 2% xylocaine 2 mL Triamcinolone: 2 mL (20 mg) + 2% xylocaine 2 mL Triamcinolone: 1 mL (40 mg) + 2% xylocaine 2 mL	NA	NA	NA	VAS	0, 10, 30 min; 1, 2, 6 h; 1, 7 days	Control (lidocaine + normal saline) group: one patient dropped out due to pain during injection
Suwannaphisit 2022 [40]	Thailand	RCT	De Quervain’s tenosynovitis	Ketorolac: 31 patients; 6 M, 25 F; median age 54.5 yrs [IQR: 10]; mean duration 30 ± 37 days Triamcinolone: 34 patients; 7 M, 27 F; median age 54 yrs [IQR: 14]; mean duration 30 ± 47.1 days	Ketorolac: 1 mL ketorolac (30 mg/mL) + 1% lidocaine 0.5 mL with adrenaline	Triamcinolone: 1 mL triamcinolone acetonide 10 mg/mL + 1% xylocaine 0.5 mL with adrenaline	Injected along the line of the tendon (proximal or distal to radial styloid, at the location of maximal pain)	Allowed to do light activities (avoid lifting weight above 10 kg); wrist motion as tolerated; oral paracetamol 500 mg as needed	NA	VAS; DASH; grip/pinch strength	6 wks	Steroid: half the patients developed some degree of hypopigmentation
Taheri 2017 [41]	Iran	RCT	Shoulder impingement syndrome	Ketorolac: 20 patients; 8 M, 12 F; mean age 49.76 ± 4.83 yrs Methylprednisolone: 20 patients; 9 M, 11 F; mean age 47.53 ± 6.92 yrs	Ketorolac: 60 mg ketorolac mixed + 2% lidocaine 1 mL	Methylprednisolone: 40 mg methylprednisolone mixed + 2% lidocaine 1 mL	Anterolateral approach	Advised to take simple analgesia as needed but to avoid oral NSAIDs	Home exercise program	VAS; CMS	1 and 3 mo	NA
Verma 2022 [51]	India	Retrospective comparative study	Knee osteoarthritis (KL grade 2 or 3)	Ketorolac: 25 patients; 11 M, 14 F; mean age 59.5 ± 10.16 yrs; mean duration 11.59 ± 4.32 mo Triamcinolone: 25 patients; 9 M, 16 F; mean age 58.6 ± 9.2 yrs; mean duration 10.46 ± 3.26 mo	Ketorolac: 10 mg of ketorolac + 0.5% lidocaine 5 mL	Triamcinolone: 40 mg of triamcinolone + 0.5% lidocaine 5 mL	Superolateral approach (three weekly injections with NSAID or steroid and, 2 weeks later, 6 mL (48 mg) of sodium hyaluronate)	Aceclofenac 100 mg BID and oral cefuroxime 250 mg BID for 3 days	NA	VAS; WOMAC; Rubin scale	1, 2, 5 wks, and 3 mo after first injection	Two patients (unspecified group) with local inflammation and pain
Xu 2020 [52]	China	Retrospective comparative study	Knee osteoarthritis (KL grade 2 or 3)	Ketorolac: 42 patients; 18 M, 24 F; mean age 59.02 ± 11.25 yrs; mean duration 8.59 ± 4.32 mo Triamcinolone: 42 patients; 16 M, 26 F; mean age 58.16 ± 10.21 yrs; mean duration 8.35 ± 3.86 mo	Ketorolac: 10 mg of ketorolac + 0.5% lidocaine 5 mL	Triamcinolone: 25 mg of triamcinolone + 0.5% lidocaine 5 mL	Superolateral approach (all patients received five weekly injections. NSAID/steroid injection was given during the first 3 weeks; for the last 2 weeks, only sodium hyaluronate (25 mg))	Imrecoxib 100 mg BID for 3 days; other analgesics not allowed	NA	VAS; WOMAC	1, 2, and 5 wks after treatment initiation, and 3 mo after the last injection	No major complications in either group NSAID injection: three patients developed mild, focal post-injection pain
Yilmaz 2019 [53]	Turkey	RCT	Knee osteoarthritis (KL grade 1 or 2)	Tenoxicam: 30 patients; 11 M, 19 F; mean age 68.07 ± 8.08 yrs; mean duration 51.03 ± 49.29 mo Triamcinolone: 30 patients; 8 M, 22 F; mean age 65.83 ± 10.13 yrs; mean duration 57.67 ± 61.69 mo	Tenoxicam: 2 mL of 20 mg	Triamcinolone: 1 mL of 20 mg	No ultrasound or fluoroscopy guidance	Advised to rest or remain immobilized and avoid any kind of weight loadings for one day; took acetaminophen as needed	NA	VAS; WOMAC	1, 3, and 6 mo	NA
Yu 2018 [42]	China	RCT	Shoulder impingement syndrome	Lornoxicam: 19 patients; 7 M,12 F; mean age 37.2 ± 10.1 yrs (range 27–63) Triclabendazole: 20 patients; 14 M, 8 F; mean age 44.7 ± 9.5 yrs (range 35–68)	Lornoxicam: 8 mg + 2% lidocaine, total 5 mL mixture	Triclabendazole: 40 mg + 2% lidocaine, total 5 mL mixture	Ultrasound-guided	NA	NA	VAS; UCLA score; ROM (abduction)	60 min, 6 wks	NA

Abbreviations: ASES, American Shoulder and Elbow Surgeons Standardized Shoulder Assessment Form; BCTQ, Boston Carpal Tunnel Syndrome Questionnaire; BID, twice a day; CMS, Constant–Murley Score; DASH, Disabilities of the Arm, Shoulder, and Hand questionnaire; ER, external rotation; F, female; GI, gastrointestinal; HHS, Harris Hip Score; HOOS, Hip Disability and Osteoarthritis Outcome Score; IR, internal rotation; KL, Kellgren–Lawrence; KOOS, Knee Injury and Osteoarthritis Outcome Score; KS, Knee Society; M, male; mo, month; NA, not available; NSAID, non-steroidal anti-inflammatory drugs; OSS, Oxford Shoulder Score; PROMIS, Patient-Reported Outcomes Measurement Information System; RCT, randomized clinical trial; RM, Roles and Maudsley; ROM, range of motion; SPADI, Shoulder Pain and Disability Index; UCLA, University of California, Los Angeles; VAS, visual analogue scale; wk, week; WOMAC, Western Ontario and McMaster Universities Arthritis Index; yr, year.

**Table 2:** Summary of main findings of included studies.

Author	Study Design	Pathology	Main Findings
Abolhasani 2019 [26]	RCT	Shoulder impingement syndrome	Ketorolac, triamcinolone, and betamethasone are equally effective in improving VAS and OSS over six weeks.
Ahn 2015 [43]	Retrospective comparative study	Adhesive capsulitis	Both groups demonstrated significant improvement in SPADI, VAS, and passive ROM at one, three, and six months. NSAID group showed greater improvement in passive external rotation and abduction at three and six months.
Aksakal 2017 [44]	RCT	Shoulder impingement syndrome	Changes in CMS and UCLA scores from baseline were higher in steroid groups at two, four, and six weeks. Steroid injection resulted in significant improvement at all follow-ups compared to previous follow-ups except for UCLA score between weeks four and six, while NSAID injection showed significant improvement from baseline to weeks two but not between weeks two and four and between weeks four and six.
Bayat 2018 [45]	RCT	Knee osteoarthritis (KL grade 2 or 3)	Pain levels (in all three questionnaires) in triamcinolone group improved more than in ketorolac group after one month. No difference was found between the groups after three months. Intra-group comparisons showed significant improvements in VAS scores and pain subscales of WOMAC and Lequesne after one and three months in both groups; improvements in general WOMAC and Lequesne scores after one and three months in triamcinolone group only.
Bellamy 2016 [46]	RCT	Knee osteoarthritis (mean KL grade 3)	Mean VAS for ketorolac and corticosteroid improved significantly from baseline at two weeks and remained improved for 24 weeks without group difference. Corticosteroids appeared to have higher WOMAC scores than ketorolac at the final follow-up. There were no significant differences in KS pain and function, Short Form 36, Tegner/Lysholm, and UCLA activity scores between the two groups throughout the 24 weeks. The cost savings per year using ketorolac instead of triamcinolone would be USD 2259.40, USD 6182.54, and USD 4159.35 for 2013, 2014, and 2015, respectively, with a total saving of USD 12,601.29 over this period.
Cift 2015 [29]	RCT	Shoulder impingement syndrome	Both groups demonstrated significant improvement in VAS, DASH, and active ROMs without significant group differences at post-treatment follow-ups. A statistically significant difference in mean change in VAS and DASH favoring NSAID injection.
Gondal 2021 [30]	RCT	Knee osteoarthritis (KL grade 2 or 3)	Both groups showed significant improvement in VAS and WOMAC at one week, one month, and three months without group difference.
Goyal 2022 [31]	RCT	Shoulder impingement syndrome	Both groups showed significant improvement in VAS and SPADI at three months without group difference. There was no significant difference in ROM at one or three months between the two groups. Two patients in the NSAID group and one patient in the steroid group required a second injection at four weeks, with one from each group showing improvement, but one patient from the NSAID group underwent arthroscopic subacromial decompression.
Guner 2013 [32]	RCT	Plantar fasciitis	Both groups showed significant improvement in terms of VAS at 6 and 12 months compared to pretreatment without group difference.
Jami 2020 [33]	RCT	Trigger finger	Both treatments were effective in improving symptoms, but the rate of improvement was better in the steroid group.
Jurgensmeier 2021 [34]	RCT	Hip and knee osteoarthritis (KL grade 2 or higher)	Both groups showed significant improvement in HOOS, KOOS, and VAS without group differences.
Karimzadeh 2023 [35]	RCT	Carpal tunnel syndrome (mild to moderate)	Both injections relieved pain, increased function, and improved electrodiagnostic findings in patients with mild to moderate carpal tunnel syndrome. Triamcinolone was more effective than ketorolac in terms of analgesic effect and improvement in symptom severity and function.
Karthikeyan 2010 [47]	RCT	Shoulder impingement syndrome	Both groups showed significant improvement in CMS at six weeks, but the improvement was higher in the steroid group. Patients in the steroid group demonstrated significantly higher improvement in the DASH and the OSS than those in the NSAID group at two, four, and six weeks.
Kim 2021 [36]	RCT	Shoulder impingement syndrome	Both groups showed significant improvements in VAS, ASES, and UCLA scores without group differences during all follow-up periods.
Kwon 2017 [48]	Retrospective comparative study	Shoulder impingement syndrome	Pain improvement was significantly greater in the steroid group at one month, but there was no significant difference in pain improvement at three months. Significant improvement in CMS without group difference. External rotation was significantly greater in the NSAID group at three months.
Leow 2018 [37]	RCT	Trigger finger	Steroid injection provided better short-term outcomes in terms of severity, resolution of triggering, and reduction in pain, swelling, and flexion deformity at 12 weeks. However, the longer-term outcomes of NSAID and steroids at 24 weeks were similar.
Matee 2021 [27]	Quasi-experimental study	Adhesive capsulitis (50%); rotator cuff syndrome (40%); shoulder impingement syndrome (10%)	Both injections resulted in significant improvement in ROM at four weeks without group difference.
Min 2013 [38]	RCT	Shoulder impingement syndrome	Both injections resulted in significant improvement of ROM and pain at four weeks, but abduction and mean improvement in the UCLA score were significantly higher in the NSAID group.
Park 2015 [49]	Retrospective comparative study	Hip osteoarthritis (KL grade 2 or 3)	Significant improvement in HHS and VAS at one, three, and six months compared to baseline without group difference.
Shakeel 2012 [39]	RCT	Trigger finger (at least grade 2 by Quinnell)	There was a significant initial improvement in Quinnell grading for the corticosteroid group compared to the NSAID group from zero to three weeks, but from three weeks to three months, the grades were ranked again, and the NSAID group did significantly better. There was no significant difference in Quinnell grading improvement between the two groups at three months.
Siddique 2021 [50]	RCT	Shoulder impingement syndrome	NSAID injections resulted in significantly lower pain scores, while steroid injections resulted in significantly higher CMS at four weeks.
Sindhupakorn 2020 [28]	RCT	Soft tissue injuries; bicipital tendinitis (21.7%); de Quervain’s tenosynovitis (31.7%); knee bursitis (4.2%); lateral epicondylitis (15%); myofascial pain syndrome (2.5%); patellar tendinitis (2.5%); plantar fasciitis (22.5%)	Both ketorolac and triamcinolone showed comparable efficacy in reducing pain intensity. Specifically, 60 mg of ketorolac demonstrated similar or even better pain reduction at several time points compared to various doses of triamcinolone. Moreover, 20 mg of triamcinolone facilitated the quickest return to normal activity post injection, with an average time of 12.35 min, while 60 mg of ketorolac had the highest success rate in returning to normal activity within 30 min.
Suwannaphisit 2022 [40]	RCT	De Quervain’s tenosynovitis	The steroid group showed higher improvement in VAS and DASH scores at six weeks. Both groups showed significant improvement in grip strength at six weeks. Furthermore, 25/31 in the ketorolac group received additional injections with triamcinolone.
Taheri 2017 [41]	RCT	Shoulder impingement syndrome	Both groups showed significant improvement in VAS and CMS at one and three months without group difference.
Verma 2022 [51]	Retrospective comparative study	Knee osteoarthritis (KL grade 2 or 3)	Both groups showed significant improvement in VAS and WOMAC at three months after the first injection without group difference.
Xu 2020 [52]	Retrospective comparative study	Knee osteoarthritis (KL grade 2 or 3)	Both groups showed significant improvement in VAS and WOMAC at three months without group difference.
Yilmaz 2019 [53]	RCT	Knee osteoarthritis (KL grade 1 or 2)	Both NSAID and steroid injection alone provided improvement in VAS and WOMAC at one month but not at three and six months. Combined NSAID and steroid injections resulted in improvement in VAS and WOMAC at one, three, and six months.
Yu 2018 [42]	RCT	Shoulder impingement syndrome	Pain improvement was seen in both interventions in both 60 min and six weeks post intervention. UCLA scores improvement was seen in both interventions in both 60 min and six weeks post intervention, but no difference was found between groups. Improvement of abduction was seen in both interventions in both 60 min and six weeks post-intervention, and no difference was found between groups at 60 min after, but NSAID group showed better recovery in shoulder abduction at six weeks after intervention.

Abbreviations: ASES, American Shoulder and Elbow Surgeons Standardized Shoulder Assessment Form; CMS, Constant–Murley Score; DASH, Disabilities of the Arm, Shoulder, and Hand Questionnaire; HHS, Harris Hip Score; HOOS, Hip Disability and Osteoarthritis Outcome Score; KOOS, Knee Injury and Osteoarthritis Outcome Score; NSAID, non-steroidal anti-inflammatory drugs; OSS, Oxford Shoulder Score; RCT, randomized clinical trial; ROM, range of motion; SPADI, Shoulder Pain and Disability Index; UCLA, University of California, Los Angeles; VAS, visual analogue scale; WOMAC, Western Ontario and McMaster Universities Arthritis Index; yr, year.

### 3.2. Study Quality and Risk of Bias Assessment

**Figure 2:**
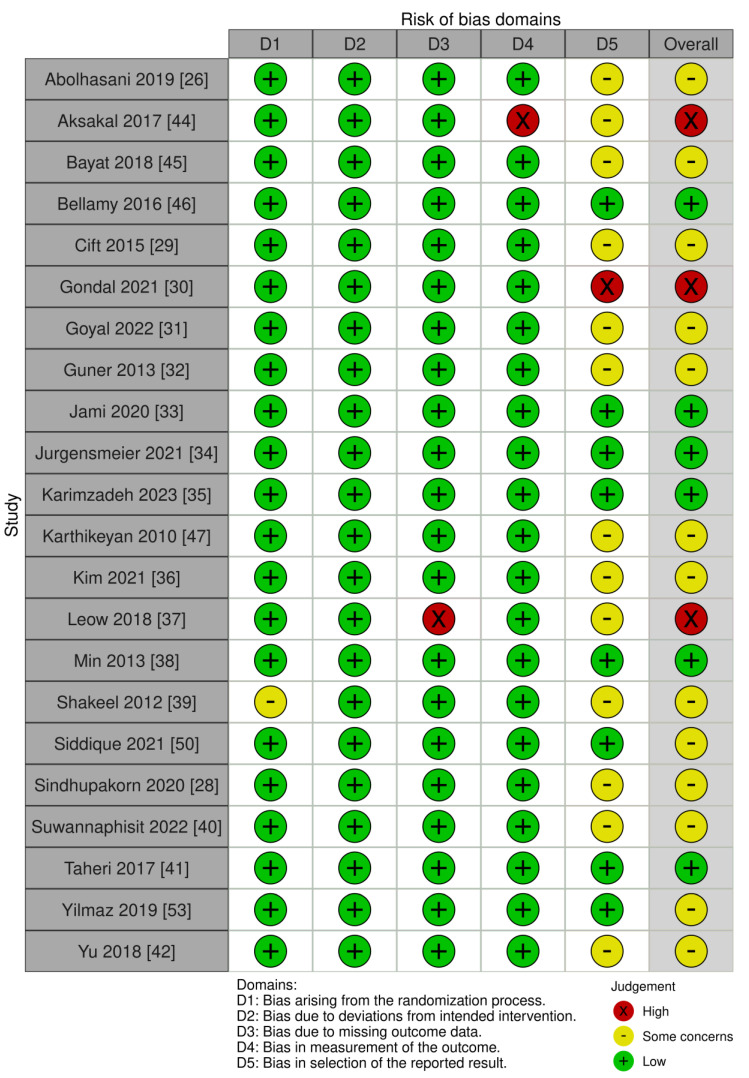
Risk of bias of included in randomized clinical trials.

Among 22 RCTs, 6 studies were deemed to have a low risk of bias, 13 studies had some concerns regarding the risk of bias, and 3 studies had a high risk of bias (Figure 2). All six non-randomized controlled trials had NOS scores of 7 to 8, indicating a low risk of bias [54].

### 3.3. Shoulder Impingement Syndrome

Eleven studies including 10 RCTs and one retrospective comparative study compared the effect of NSAID injections against steroid injections. Two RCTs [38,41] were deemed to have a low risk of bias, seven RCTs had some concerns regarding the risk of bias [26,29,31,36,42,47,50], and one RCT [44] had a high risk of bias. One retrospective comparative study was deemed to have a low risk of bias [48]. For NSAID injections, 30–60 mg of ketorolac, 8 mg of lornoxicam, and 20 mg of tenoxicam were used. For steroid injections, 40–80 mg of triamcinolone and 6–7 mg of betamethasone were utilized. No adverse events were reported in six studies [31,41,42,44,47,48]. Two cases of temporary hypotension were observed in the tenoxicam group [29], while one case of uncontrolled diabetes, two cases of facial flushing [36], and one case of a self-resolved fainting episode [38] were reported with the triamcinolone injection.

**Figure 3:**
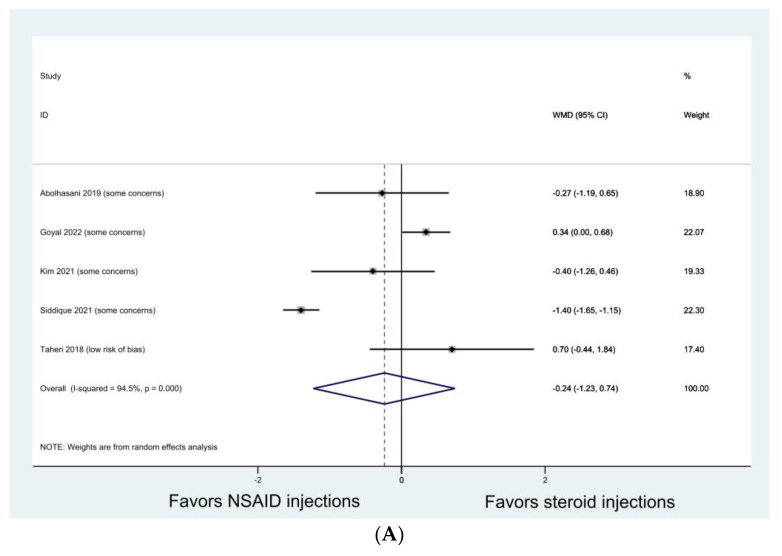

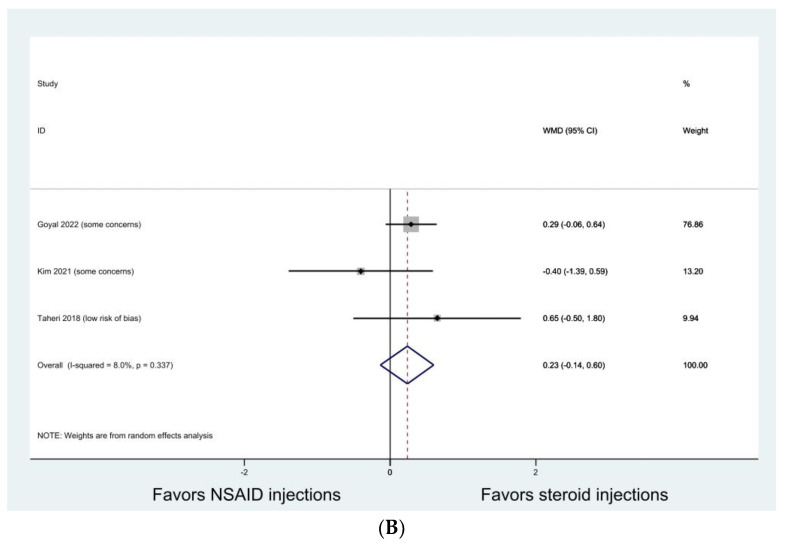
Meta-analyses comparing NSAID injection with steroid injection in pain visual analogue scale (VAS) for shoulder impingement syndrome. (**A**) VAS at 1 month; (**B**) VAS at 3 months.

In nine studies that reported VAS [26,29,31,36,38,41,42,48,50], both NSAID injections and steroid injections significantly improved pain at the end of their follow-ups. Our meta-analysis of five studies [26,31,36,41,50] demonstrated that there was no significant difference in pain VAS between the NSAID injection and the steroid injection at 1 month (Figure 3A, WMD −0.244; 95% CI, −1.232 to 0.745; I^2^, 94.5%). Our meta-analysis of three studies [31,36,41] demonstrated that there was no significant difference in pain VAS between the NSAID injection and the steroid injection at 3 months (Figure 3B, WMD 0.235; 95% CI, −0.135 to 0.604; I^2^, 8%).

In five studies that reported shoulder range of motion (ROM) [29,31,38,42,48], both NSAID injections and steroid injections improved ROM at the end of their follow-ups. In two RCTs, the NSAID group showed better recovery in shoulder abduction at 4 weeks [38] and 6 weeks [42]. However, there were no significant differences between NSAID injections and steroid injections in ROM including flexion, abduction, internal rotation, and external rotation in three other studies during follow-ups [29,31,48].

In five studies that reported the Constant–Murley Score (CMS) [41,44,47,48,50], both NSAID injections and steroid injections significantly improved the scores at the end of their follow-ups, but one study [47] reported greater improvement with the methylprednisolone injection at 6 weeks, while another study [50] observed greater improvement with the ketorolac injection at 1 month.

In four studies that reported the University of California Los Angeles (UCLA) shoulder score [36,38,42,44], both NSAID injections and steroid injections significantly improved the scores at the end of their follow-ups, but one study [44] reported greater improvement with betamethasone at 2, 4, and 6 weeks, while another study [38] showed greater improvement with ketorolac at 4 weeks. 

In the two studies [29,47] that reported Disabilities of Arm, Shoulder, and Hand (DASH) scores, both NSAID injections and steroid injections improved the scores at the end of their follow-ups, with one study [47] reporting greater improvement with methylprednisolone at 2, 4, and 6 weeks.

In two studies [26,47] that reported the Oxford Shoulder Score, both NSAID injections and steroid injections improved the scores over 6 weeks, but one study showed that methylprednisolone resulted in significantly greater improvement in the OSS at 2, 4, and 6 weeks.

One study [31] reported the Shoulder Pain and Disability Index score (SPADI) and another study [36] reported the American Shoulder and Elbow Surgeons (ASES) score, and both NSAID injections and steroid injections improved these outcomes at 3 months without difference between the injections.

### 3.4. Knee Osteoarthritis

Five RCTs [30,34,45,46,53] and two retrospective comparative studies [51,52] compared the effect of NSAID injections with steroid injections in knee osteoarthritis. Two RCTs [34,46] were deemed to have a low risk of bias, two RCTs [45,53] had some concerns regarding the risk of bias, and one RCT [30] had a high risk of bias. Two retrospective comparative studies [51,52] were deemed to have a low risk of bias. For the NSAID injection, 30 mg of ketorolac was used in three studies [34,45,46]. In one RCT, 30 mg of ketorolac was mixed with 25 mg sodium hyaluronate [30], and in another RCT, 20 mg of tenoxicam was used [53]. In one study, three weekly injections with 10 mg of ketorolac were given followed by 48 mg of sodium hyaluronate 2 weeks after the third ketorolac injection [51], while in another study, a total of five weekly injections were given with 10 mg ketorolac for the first 3 weeks followed by 25 mg of sodium hyaluronate for the remaining 2 weeks [52]. For the steroid injection, triamcinolone in 20 mg [53], 40 mg [45], 80 mg [34,46], and 80 mg dosages in conjunction with 25 mg of sodium hyaluronate [30] were used. In one study, three weekly injections with 40 mg of triamcinolone were given, followed by 48 mg of sodium hyaluronate 2 weeks after the third triamcinolone injection [51], while in another study, a total of five weekly injections were given with 25 mg of triamcinolone for the first 3 weeks, followed by 25 mg of sodium hyaluronate for the remaining 2 weeks [52]. Three studies did not report adverse events [30,45,46]. In one RCT [34], in the NSAID group, one patient developed a headache and nausea which resolved the next day, and another patient developed GI bleeding, but this occurred in the setting of an increased warfarin dose around 2 months after the ketorolac injection. In the steroid group, hyperglycemia was noted in two diabetic patients during the first week post injection, and the cases of nausea, hypertension, flare of temporal arteritis were each recorded once. Two patients (unspecified group) developed local inflammation and pain in one study [51], and three patients developed mild, focal postinjection pain in the NSAID group in another study [52]. No major complications were observed in one RCT [53].

**Figure 4:**
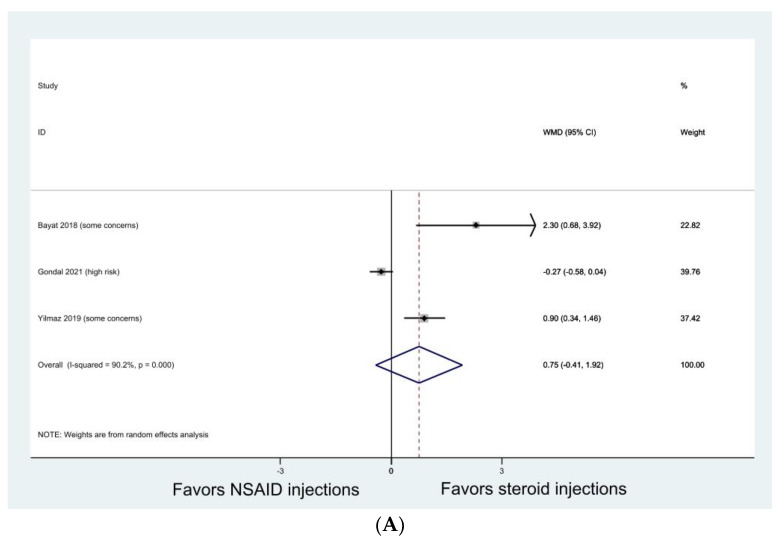

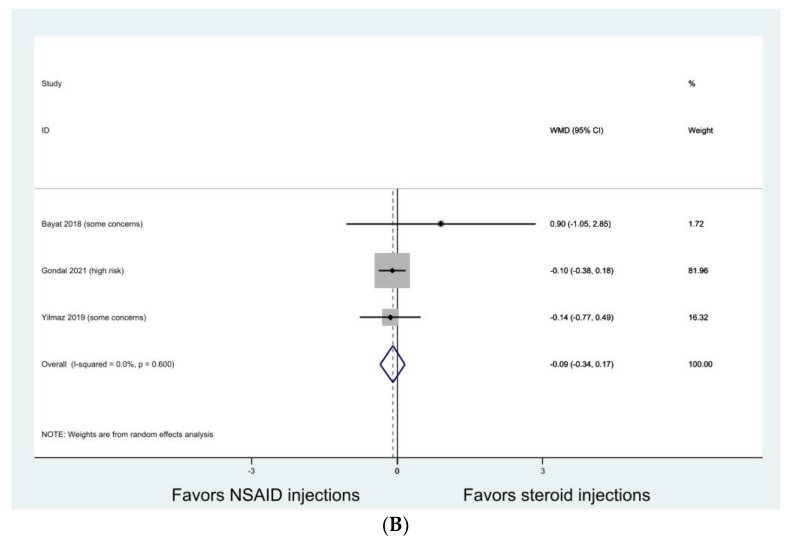
Meta-analyses comparing NSAID injection with steroid injection in pain visual analogue scale (VAS) for knee osteoarthritis. (**A**) VAS at 1 month; (**B**) VAS at 3 months.

In seven studies [30,34,45,46,51,52,53] that reported VAS, all studies showed that both NSAID injections and steroid injections significantly improved VAS at the end of their follow-ups, except for one study [53] which demonstrated that the effects of both injections diminished at 3 and 6 months. Our meta-analysis of three studies [30,45,53] demonstrated that there was no significant difference between the NSAID injection and the steroid injection in pain VAS at 1 month (Figure 4A, WMD 0.75; 95% CI, −0.41 to 1.92; I^2^, 90.2%) and 3 months (Figure 4B, WMD −0.089; 95% CI, −0.345 to 0.166; I^2^, 0%).

For six studies [30,45,46,51,52,53] that reported WOMAC, all studies showed that both NSAID injections and steroid injections improved WOMAC scores at the end of their final follow-ups, except for two studies which favored steroids injections [45,46] and one study [53] which observed that the effects of both injections disappeared at 3 and 6 months.

### 3.5. Hip Osteoarthritis

One RCT [34] with a low risk of bias and one retrospective comparative study [49] with low risk of bias compared the effect of an NSAID injection with a steroid injection in hip osteoarthritis. Both studies used 30 mg of ketorolac, while the dosage of triamcinolone in the RCT was 80 mg, and in the other study, it was 40 mg. In the RCT, both groups showed improvement in the Hip Disability and Osteoarthritis Outcome Score and VAS scores compared to baseline at 1 week, 1 month, and 3 months without group differences [34]. In this study, the authors did not report adverse events. In the retrospective comparative study, the Harris Hip Score and VAS improved at 1, 3, and 6 months in both groups without group differences [49]. In this study, the authors observed four local adverse events described as a mild, transient sensation of pain and heaviness in the joint injected with the ketorolac.

### 3.6. Trigger Finger

Three RCTs compared the effect of NSAID injections with steroid injections in trigger finger. Two RCTs [33,39] had some concerns for risk of bias, and one RCT [37] had a high risk of bias. Due to the heterogeneity of the outcome measures, a meta-analysis was not performed, but all three RCTs reported a faster recovery associated with steroid injections. In one RCT, the mean change in Quinnell grading from the initiation of treatment to the 6th week and to 12 months was faster in the group receiving 20 mg of methylprednisolone compared to the group receiving 12.5 mg of diclofenac [33]. The authors did not report adverse events. In another RCT, 5 mg triamcinolone injections provided better short-term outcomes than 15 mg ketorolac injections in terms of severity, resolution of triggering, swelling, and flexion deformity, and reduction in pain at 12 weeks, although the longer-term outcomes at 24 weeks were similar [37]. No adverse events were observed in either group. In the other RCT, there was a significant initial improvement in Quinnell grading for the group receiving 20 mg of triamcinolone compared to the group receiving 12.5 mg of diclofenac from 0 to 3 weeks, but from 3 weeks to 3 months, repeat grading showed the NSAID group did significantly better. No significant difference in Quinnell grading improvement between the two groups was seen at 3 months [39]. In the diclofenac group, the adverse events included two patients (4%) with continuous pain at the injection site, three patients (6%) with nodular swelling at the injection site, two patients (4%) with stiffness of the injected finger, and one patient (2%) with the recurrence of triggering pain. In the triamcinolone group, the adverse events included one patient (2%) with continuous pain at the injection site and nine patients (18%) with the recurrence of triggering pain.

### 3.7. Adhesive Capsulitis

One retrospective comparative study with a low risk of bias compared the effect of a 30 mg ketorolac injection plus capsular distension with a 40 mg triamcinolone injection (without capsular distension) [43]. The authors found that both groups demonstrated significant improvement in the SPADI, VAS, and passive ROM at 1, 3, and 6 months. Two cases of dizziness and transient muscle weakness were reported in the ketorolac group and two cases of steroid-induced synovitis in the triamcinolone group, but there were no other severe complications.

### 3.8. Plantar fasciitis

One RCT with some concerns for risk of bias compared the effect of a 10 mg tenoxicam injection with a 40 mg methylprednisolone in patients with plantar fasciitis [32]. The study found that both groups showed significant improvement in VAS at 6 and 12 months compared to pretreatment without group differences. No adverse events were observed in either group.

### 3.9. Carpal Tunnel Syndrome

One RCT with a low risk of bias compared the effect of a 30 mg ketorolac injection with a 40 mg triamcinolone injection in carpal tunnel syndrome [35]. The study found that both injections relieved pain, increased function, and improved electrodiagnostic findings in patients with mild to moderate carpal tunnel syndrome at 3 months. However, triamcinolone injection was more effective than ketorolac in terms of analgesic effect and improvement in symptom severity and function. Seven patients from each group experienced adverse events such as a warm sensation, stiffness, and heaviness at the injection site without significant group differences.

### 3.10. De Quervain’s Tenosynovitis

One RCT with some concerns for risk of bias compared the effect of 30 mg of ketorolac with 10 mg of triamcinolone in patients with de Quervain’s tenosynovitis [40]. The study found that patients who received triamcinolone injections showed greater improvement in VAS and DASH scores at 6 weeks. Furthermore, 25 out of 31 patients in the ketorolac group needed additional injections with triamcinolone at 6 weeks, while no one in the triamcinolone group did. No major adverse events were reported, but half of the patients in the triamcinolone group developed some degree of hypopigmentation.

### 3.11. Other Soft Tissue Injuries

Two studies compared NSAID injections with steroid injections in multiple soft tissue conditions. Matee et al. conducted a quasi-experimental study with a low risk of bias including patients with adhesive capsulitis (50%), rotator cuff syndrome (40%), and shoulder impingement syndrome (10%) and compared 30 mg of ketorolac with 40 mg of methylprednisolone [27]. The authors found that both injections resulted in significant improvement in ROM (flexion, extension abduction, internal rotation, and external rotation) without group differences. The authors did not report adverse events in this study. Sindhupakorn et al. conducted an RCT with some concerns for risk of bias including patients with bicipital tendinitis (21.7%), de Quervain’s tenosynovitis (31.7%), knee bursitis (4.2%), lateral epicondylitis (15%), myofascial pain syndrome (2.5%), patellar tendinitis (2.5%), and plantar fasciitis (22.5%) [28]. The authors compared 30–60 mg of ketorolac with 10–40 mg of triamcinolone and found that ketorolac in 30 mg and 60 mg dosages and 10 mg of triamcinolone were non-inferior to 40 mg of triamcinolone. No adverse events were noted in the groups receiving either ketorolac or triamcinolone.

## 4. Discussion

The purpose of this review was to summarize the available literature comparing NSAID injections with steroid injections in upper and lower extremity musculoskeletal conditions. The results of our meta-analyses suggest that NSAID injections may be as effective as corticosteroids in pain reduction in shoulder impingement syndrome and knee osteoarthritis. Based on the few studies included, NSAID injections may be as effective as corticosteroids in hip osteoarthritis, adhesive capsulitis, and plantar fasciitis as well. On the other hand, in trigger finger, de Quervain’s tenosynovitis, and carpal tunnel syndrome, steroid injections were shown to offer faster or better pain relief in few studies.

For intraarticular injections, steroids are considered one of the first-line injectable agents for persistent pain. However, in vitro experiments demonstrate decreased cell viability of chondrocytes [55] after exposure to methylprednisolone, and in rabbit models, cartilage necrosis was observed with a corticosteroid injection [56]. Clinically, cases of osteonecrosis after intraarticular hip injections have been reported [10,11,12], and there may be an increased risk of knee radiographic osteoarthritis progression with intraarticular injections, especially with repeated injections [57]. Further, for patients receiving epidural steroid injections, decreased bone mineral density (BMD) has been observed with increased risk of vertebral fracture [7]. For the management of tendinopathies, despite the short-term efficacy [58], clinicians tend to minimize using steroids given the risks of tendon rupture or atrophy [8,9]. Hyperglycemia is another potential side effect to consider when injecting steroids in diabetic patients [13]. Given these risks, patients who require repetitive intraarticular injections for pain control, have low BMD or diabetes, and suffer from chronic tendinopathies may need alternatives to steroid injections in order to reduce pain and improve function.

Based on the results of our review, NSAID injections can be a viable alternative to intraarticular steroid injections and steroid injections for certain musculoskeletal disorders. In fact, NSAID injections may have some advantages over steroids as well as other interventions. While the results of animal and in vitro studies are mixed [55,56,59,60,61,62,63,64,65], some studies [55,56,59,63,64] showed NSAID injections or exposure to NSAID did not affect cartilage, synovium, or chondrocyte viability. Considering these findings, intraarticular NSAID injections may be theoretically safer than steroid injections in the long term. In addition, single doses of intraarticular NSAIDs appear to provide far less total systemic exposure compared to a one-week course of oral NSAIDs but higher maximum concentrations to the synovium [18], findings which suggest that systemic adverse events associated with intraarticular NSAID injections be less likely to occur than with oral NSAIDs.

Furthermore, even though it is widely accepted that chronic tendinopathy is a degenerative process, there is convincing evidence that the inflammatory response is also involved in the pathogenesis [66,67]. Therefore, peritendinous NSAID injections may be helpful in tendinopathies in which steroid injections are not desirable. Lastly, while platelet-rich plasma (PRP) [68], extracorporeal shockwave therapy (ESWT) [69], and prolotherapy [70] are some of the common interventions concomitantly used with physical therapy to enhance recovery in musculoskeletal injuries, these treatments are often not covered by insurance and incur out-of-pocket costs. Given that the cost associated with NSAID use is less than corticosteroids such as methylprednisolone, betamethasone, and triamcinolone [20], NSAID injections may represent the cheapest option among other interventions. Activity restrictions following PRP injections [71] and the three to five sessions needed for ESWT [72] may be potential limiting factors for these treatments as well.

### 4.1. Clinical Implications and Future Research Direction

When considering injection therapies for osteoarthritis, NSAID injections may be discussed along with steroid injections, especially in knee and hip osteoarthritis. For those patients who may require frequent injections, NSAID injections can serve as a bridge between steroid injections to prolong the interval between two steroid injections. NSAID injections can also be considered while awaiting arthroplasty because steroid injections within 3 months of total knee arthroplasty were found to increase periprosthetic joint infection [73]. Further clinical trials would be needed to investigate whether the intraarticular injection of NSAIDs is safe prior to arthroplasty and effective in other joints including shoulder, elbow, foot, and ankle joints. Moreover, NSAID injections may be combined with steroids as one of the RCTs included in our review demonstrated better results at 3 and 6 months with the combined therapy of triamcinolone and tenoxicam compared to triamcinolone or tenoxicam alone in knee osteoarthritis [53].

In patients with shoulder impingement syndrome, an NSAID injection can also be an effective and safe choice. Our results are consistent with a previous systematic review which focused on shoulder impingement syndrome and found that NSAID injections are as effective as steroid injections for short-term pain reduction and functional improvement [20]. Given that steroid injections are associated with an increased risk of rotator cuff tears in a temporal and dose-dependent manner [74], when patients present with concurrent rotator cuff tear, NSAID injections may be preferred to steroids.

Currently, there is lack of research investigating the effect of NSAID injections in soft tissue injuries including tendinopathies and fasciopathies. A few studies included in our review showed promising results in lateral epicondylitis [28], biceps tendinopathy [28], patellar tendinopathy [28], and plantar fasciitis [32]. Although animal studies have suggested that NSAIDs may interfere with tendon healing [75], more human research would be needed to confirm the efficacy and safety of NSAID injections in tendinopathies and other soft tissue conditions. This will be particularly helpful for those patients who may not be able to afford other interventions such as PRP, prolotherapy, or ESWT which require out-of-pocket costs.

While NSAID injections may be useful in certain conditions as described above, it is worth noting that in trigger finger, de Quervain’s tenosynovitis, and carpal tunnel syndrome, steroid injections appeared to have superior outcomes over NSAID injections. One of the possible explanations for this finding is that steroid injections not only have an anti-inflammatory effect but also affect the connective tissue and the surrounding peritendinous tissues by reducing collagen production and the formation of extracellular matrix molecules and granulation tissues [76]. Furthermore, both trigger finger and de Quervain’s tenosynovitis result from stenosing tenosynovitis which may require more aggressive interventions to manage symptoms.

The types and dosage of NSAIDs varied across the studies. Ketorolac was most commonly used, followed by tenoxicam, diclofenac, lornoxicam, and indomethacin. While the types of NSAIDs and doses used in the studies demonstrated no significant differences in outcomes with different types of steroids, future clinical trials evaluating different types or doses of NSAIDs would be necessary to determine the optimal medication and dosage.

Another consideration while designing RCTs to show NSAID injections are not worse than steroid injections would be to set an appropriate non-inferiority margin as well as to perform adequate power analysis. In some RCTs included in our review, a power analysis and sample size calculations were missing details or not clearly reported, [29,32,39,41,42,50], or non-inferiority margins for primary outcomes were not reported [34]. Without this information, it would be difficult to distinguish whether these trials were underpowered to detect differences or their results demonstrated non-inferiority. Future RCTs would need to clearly define non-inferiority margins and perform adequate power analyses to demonstrate the non-inferiority of NSAID injections.

### 4.2. Limitations

Our study has several limitations. First, meta-analyses were performed with small numbers of RCTs in an exploratory nature and were not conducted for certain outcomes in shoulder impingement syndrome due to missing means or standard deviations and for trigger finger because of the heterogeneous outcomes reported. Also, given the small number of studies included in the meta-analyses, Grading of Recommendations Assessment, Development and Evaluation (GRADE) was not performed. However, one of the aims of this review was to provide an overview of the available literature and propose a research direction based on the existing studies. Second, except for shoulder impingement syndrome and knee osteoarthritis, only a small number of studies were included for the remaining conditions, and therefore, the results of conditions such as adhesive capsulitis, plantar fasciitis, and soft tissue conditions should be interpreted with caution. The findings of this review should be further substantiated by more high-quality RCTs. Third, while publication bias was not assessed given the small numbers of studies included, there may be a risk of publication bias. Lastly, while ketorolac and triamcinolone were most commonly used, the dosage and types of NSAIDs and steroids differed across the studies, and therefore, the results of certain NSAID injections or certain conditions may not be generalized to other conditions. The optimal type and dosage need to be further explored in future studies.

## 5. Conclusions

Our systematic review with a meta-analysis showed that NSAID injections may be potential alternatives to steroid injections without major complications, especially in shoulder impingement syndrome and knee osteoarthritis. Based on the results from a few studies, NSAID injections may also be as effective as corticosteroids in hip osteoarthritis, adhesive capsulitis, and plantar fasciitis. However, further evidence is needed to substantiate these claims, and more high-quality RCTs would be warranted to expand indications in other conditions and identify the optimal type and dosage.

## Data Availability

Data available on request from the authors.

## Supplementary Materials

The following supporting information can be downloaded at: https://www.mdpi.com/article/10.3390/jcm13041132/s1, Table S1: Search strategy.
