# Supplementary material for: Nonsteroidal Anti-Inflammatory Drug Injections versus Steroid Injections in the Management of Upper and Lower Extremity Orthopedic Conditions: A Systematic Review with Meta-Analysis
Source: J Clin Med. 2024 Feb 17;13(4):1132. doi: 10.3390/jcm13041132 (PMC10889729; doi:10.3390/jcm13041132)
Supplement: Supplementary file 1 [file jcm-13-01132-s001.zip › jcm-2848684-supplementary.pdf]

Supplementary Material

Table S1. Search strategy

Table S1. Search Strategy.

**PUBMED (NLM)**	(("GLUCOCORTICOIDS"[MESH TERMS] OR "STEROIDS"[MESH TERMS] OR "STEROID*"[TITLE/ABSTRACT] OR "CORTICOSTEROID*"[ALL FIELDS] OR "TRIAMCINOLONE"[TITLE/ABSTRACT] OR "KENALOG"[TITLE/ABSTRACT] OR "BETAMETHASONE"[TITLE/ABSTRACT] OR "METHYLPREDNISOLONE"[TITLE/ABSTRACT] OR "DEPO-MEDROL"[TITLE/ABSTRACT] OR "DEXAMETHASONE"[TITLE/ABSTRACT]) AND ("INJECT*"[TITLE/ABSTRACT] OR "INJECTIONS"[MESH TERMS]) AND ("ANTI INFLAMMATORY AGENTS, NON STEROIDAL"[MESH TERMS] OR ((("ANTI INFLAMMATORY AGENTS NON STEROIDAL"[PHARMACOLOGICAL ACTION] OR "NON STEROIDAL ANTI INFLAMMATORY AGENT*"[TITLE/ABSTRACT] OR "NONSTEROIDAL ANTIINFLAMMATORY AGENT*"[TITLE/ABSTRACT] OR "NONSTEROIDAL ANTI INFLAMMATORY AGENT*"[TITLE/ABSTRACT] OR "NON STEROIDAL ANTIINFLAMMATORY AGENT*"[TITLE/ABSTRACT] OR "NSAID*"[TITLE/ABSTRACT]) AND "KETOROLAC"[TITLE/ABSTRACT]) OR "TORADOL"[TITLE/ABSTRACT] OR "TENOXICAM"[TITLE/ABSTRACT] OR "LORNOXICAM"[TITLE/ABSTRACT] OR "INDOMETHACIN"[TITLE/ABSTRACT])) AND (("MUSCULOSKELETAL DISEASES"[MESH TERMS] OR "BONE DISEASES"[MESH TERMS] OR "JOINT DISEASES"[MESH TERMS] OR ("MUSCULOSKELETAL SYSTEM"[MESH TERMS] OR "ELBOW"[MESH TERMS] OR "ANKLE"[MESH TERMS] OR "FOOT"[MESH TERMS] OR "HIP"[MESH TERMS] OR "KNEE"[MESH TERMS] OR "SHOULDER"[MESH TERMS] OR "WRIST"[MESH TERMS] OR "BONE*"[TITLE/ABSTRACT] OR "JOINT*"[TITLE/ABSTRACT] OR "MUSCULOSKELETAL*"[TITLE/ABSTRACT] OR "SKELET*"[TITLE/ABSTRACT] OR "SHOULDER*"[TITLE/ABSTRACT] OR "ELBOW*"[TITLE/ABSTRACT] OR "WRIST*"[TITLE/ABSTRACT] OR "FINGER*"[TITLE/ABSTRACT] OR "HIP"[TITLE/ABSTRACT] OR "HIPS"[TITLE/ABSTRACT] OR "KNEE*"[TITLE/ABSTRACT] OR "ANKLE*"[TITLE/ABSTRACT] OR "FOOT"[TITLE/ABSTRACT] OR "FEET*"[TITLE/ABSTRACT] OR "ORTHOPEDIC*"[TITLE/ABSTRACT] OR "ORTHOPAEDIC*"[TITLE/ABSTRACT] OR "LIGAMENT*"[TITLE/ABSTRACT] OR "PLANTAR*"[TITLE/ABSTRACT] OR "MENISCUS"[TITLE/ABSTRACT])) AND ("DISEASE*"[ALL FIELDS] OR "DISORDER*"[ALL FIELDS] OR "CONDITION*"[ALL FIELDS] OR ("SYNDROM*"[ALL FIELDS] AND "OR INJUR*"[TITLE/ABSTRACT]) OR "ARTHRITI*"[TITLE/ABSTRACT] OR "TENDINOPATH*"[TITLE/ABSTRACT] OR "FASCIITIS"[TITLE/ABSTRACT] OR "IMPINGEMENT*"[TITLE/ABSTRACT] OR "BURSITIS"[TITLE/ABSTRACT] OR "OSTEOARTHRITI*"[TITLE/ABSTRACT] OR "FRATURE*"[TITLE/ABSTRACT]))))	**629**

**EMBASE (ELSEVIER)**	**(**(NSAID* OR “nonsteroidal anti-inflammatory drugs” OR ketorolac OR Toradol OR tenoxicam OR Tilcotil OR lornoxicam OR Xefo):ti,ab,kw AND (steroids OR corticosteroids OR glucocorticoids OR triamcinolone OR Kenalog OR betamethasone OR Celestone OR methylprednisolone OR Depo-Medrol OR dexamethasone OR Decadron OR hydrocortisone OR Cortef OR prednisolone OR Prelone OR prednisone OR Deltasone):ti,ab,kw AND (inject* OR “local injection” OR “intra-articular injection”):ti,ab,kw<br>NOT (oral OR tablet* OR capsule*)):ti,ab,kw AND (((musculoskeletal condition* OR musculoskeletal disorder* OR musculoskeletal disease* OR musculoskeletal problem* OR musculoskeletal injur* OR musculoskeletal pain) OR (“joint pain” OR “joint inflammation” OR “joint stiffness” OR “joint swelling” OR “ joint damage” OR “joint degeneration” OR “joint injury”) OR (Shoulder OR elbow OR wrist OR finger OR spine OR back OR hip OR knee OR ankle OR foot OR toe) OR (arthritis) OR (osteoarthritis OR “degenerative joint disease” OR DJD OR “wear and tear arthritis”) OR (back pain) OR (low back pain) OR (neck pain) OR (bursitis OR “bursal inflammation” OR “bursal injury” OR “bursal pain” OR “bursal swelling”) OR (impingement OR compression OR pinching) OR (capsulitis OR “capsule inflammation” OR “capsule injury” OR “capsule pain” OR “capsule stiffness”) OR (tendinitis OR tenosynovitis OR tendinopathy OR tendinosis) OR (plantar fasciitis OR heel spur syndrome OR fasciitis OR “fascia inflammation” OR “fascia injury” OR “fascia pain” OR fasicopathy))**)**:ti,ab,kw	516
**COCHRANE**	((“Anti-Inflammatory Agents, Non-Steroidal”[Mesh] OR NSAID* OR “nonsteroidal anti-inflammatory drugs” OR ketorolac OR Toradol OR tenoxicam OR Tilcotil OR lornoxicam OR Xefo) **AND**<br>(“Adrenal Cortex Hormones”[Mesh] OR steroids OR corticosteroids OR glucocorticoids OR triamcinolone OR Kenalog OR betamethasone OR Celestone OR methylprednisolone OR Depo-Medrol OR dexamethasone OR Decadron OR hydrocortisone OR Cortef OR prednisolone OR Prelone OR prednisone OR Deltasone) **AND** (“Injections”[Mesh] OR inject* OR “local injection” OR “intra-articular injection”)<br>NOT (“Administration, Oral”[Mesh] OR oral OR tablet* OR capsule*)) **AND**<br>((“Musculoskeletal Diseases”[Mesh] musculoskeletal condition* OR musculoskeletal disorder* OR musculoskeletal disease* OR musculoskeletal problem* OR musculoskeletal injur* OR musculoskeletal pain) OR<br>(“joint pain” OR “joint inflammation” OR “joint stiffness” OR “joint swelling” OR “ joint damage” OR “joint degeneration” OR “joint injury”) OR (Shoulder OR elbow OR wrist OR finger OR spine OR back OR hip OR knee OR ankle OR foot OR toe) OR (“Arthritis”[Mesh] arthritis) OR (“Osteoarthritis”[Mesh] osteoarthritis OR “degenerative joint disease” OR DJD OR “wear and tear arthritis”)<br>OR (“Back Pain”[Mesh] back pain) OR (“Low Back Pain”[Mesh] low back pain) OR (“Neck Pain”[Mesh] neck pain) OR (“Bursitis”[Mesh] bursitis OR “bursal inflammation” OR “bursal injury” OR “bursal pain” OR “bursal swelling”) OR (impingement OR compression OR pinching) OR (capsulitis OR “capsule inflammation” OR “capsule injury” OR “capsule pain” OR “capsule stiffness”) OR (“Tendinopathy”[Mesh] tendinitis OR tenosynovitis OR tendinopathy OR tendinosis) OR (“Fasciitis, Plantar”[Mesh] plantar fasciitis OR heel spur syndrome OR fasciitis OR “fascia inflammation” OR “fascia injury” OR “fascia pain” OR fasicopathy))	647

**WEB OF SCIENCE**	(ALL=(NSAID*) OR ALL=("nonsteroidal anti-inflammatory drugs") OR ALL=(ketorolac) OR ALL=(Toradol) OR ALL=(tenoxicam) OR ALL=(Tilcotil) OR ALL=(lornoxicam) OR ALL=(Xefo)) AND (ALL=(steroids) OR ALL=(corticosteroids) OR ALL=(glucocorticoids) OR ALL=(triamcinolone) OR ALL=(Kenalog) OR ALL=(betamethasone) OR ALL=(Celestone) OR ALL=(methylprednisolone) OR ALL=(Depo-Medrol) OR ALL=(dexamethasone) OR ALL=(Decadron) OR ALL=(hydrocortisone) OR ALL=(Cortef) OR ALL=(prednisolone) OR ALL=(Prelone) OR ALL=(prednisone) OR ALL=(Deltasone)) AND ((ALL=(inject*) OR ALL=(“local injection”) OR ALL=(“intra-articular injection”)) NOT (ALL=(oral) OR ALL=(tablet*) OR ALL=(capsule*))) AND (ALL=(musculoskeletal condition*) OR ALL=(musculoskeletal disorder*) OR ALL=(musculoskeletal disease*) OR ALL=(musculoskeletal problem*) OR ALL=(musculoskeletal injur*) OR ALL=(musculoskeletal pain) OR ALL=(“joint pain”) OR ALL=(“joint inflammation”) OR ALL=(“joint stiffness”) OR ALL=(“joint swelling”) OR ALL=(“ joint damage”) OR ALL=(“joint degeneration”) OR ALL=(“joint injury”) OR ALL=(Shoulder) OR ALL=(elbow) OR ALL=(wrist) OR ALL=(finger) OR ALL=(spine) OR ALL=(back) OR ALL=(hip) OR ALL=(knee) OR ALL=(ankle) OR ALL=(foot) OR ALL=(toe) OR ALL=(arthritis) OR ALL=(osteoarthritis) OR ALL=(“degenerative joint disease”) OR ALL=(DJD) OR ALL=(“wear and tear arthritis”) OR ALL=(“back pain”) OR ALL=(“low back pain”) OR ALL=(“neck pain”) OR ALL=(bursitis) OR ALL=(“bursal inflammation”) OR ALL=(“bursal injury”) OR ALL=(“bursal pain”) OR ALL=(“bursal swelling”) OR ALL=(impingement) OR ALL=(compression) OR ALL=(pinching))	498
